# Nutritional condition affects tergal gland secretion and courtship success of male cockroaches

**DOI:** 10.1371/journal.pone.0271344

**Published:** 2022-08-03

**Authors:** Samantha McPherson, Ayako Wada-Katsumata, Eduardo Hatano, Jules Silverman, Coby Schal

**Affiliations:** Department of Entomology and Plant Pathology, North Carolina State University, Raleigh, North Carolina, United States of America; Indian Institute of Science, INDIA

## Abstract

An integral part of the courtship sequence of the German cockroach (*Blattella germanica*) involves the male raising his wings to expose tergal glands on his dorsum. When a female cockroach feeds on the secretion of these glands, she is optimally positioned for mating. Core chemical components have been identified, but the effect of male diet on the quality of the tergal gland secretion remains unexplored. After validating the pivotal role of tergal feeding in mating, we starved or fed reproductively mature males for one week. We then paired each male with a sexually receptive female and observed their interactions through an infrared-sensitive camera. While starvation had no effect on male courtship behavior, it did influence the duration of female tergal feeding and mating outcomes. Females fed longer on the gland secretion of fed males, and fed males experienced greater mating success than starved males (73.9% vs. 48.3%, respectively). These results suggest that the quality of the tergal gland secretions, and by association mating success, are dependent on the nutritional condition of the male.

## Introduction

In “*The Origin of Species*” [1], Charles Darwin coined the term sexual selection and in “*The Descent of Man and Selection in Relation to Sex*” [2] he further developed the concept to account for traits that secure a mate, but have no adaptive value for survival. Since then, the balancing of adaptive traits that maximize reproductive success with those that ensure individual survival has been a much-studied area across many taxa. One common sexually selected trait is nuptial gift giving, where one sex, usually the male, offers to a potential mate a resource to lower her behavioral threshold of sexual receptivity. Alternatively, the gift may be presented after a sexual encounter to ensure paternity and/or maximize the health of his offspring [3]. Pre- and post-nuptial gifts are especially prominent in insects and other arthropods.

A nutritional nuptial gift may be either an acquired food resource, such as a prey item, or a somatic gift that an animal produces [4]. For example, some species of spiders and hangingflies (Mecoptera: Bittacidae) employ the food strategy by offering the female a prey item to ensure longer copulation and prevent cannibalism [5, 6]. An extreme somatic strategy is when a male offers his own body as a nutritional resource (sexual cannibalism), as does the praying mantis, where the male continues mating with a female that is actively consuming him [7]; the male Australian redback spider (*Lactrodectus hasseti*) employs a similar strategy by somersaulting himself into the mouthparts of the female post copulation [8].

More common somatic offerings are spermatophores that serve for sperm delivery as well as post-copulatory gifts, sometimes accompanied by components that males produce solely as nuptial gifts. For example, some orthopterans such as bush crickets (Tettigoniidae) and decorated crickets, *Gryllodes sigillatus* (Gryllidae) produce spermatophylaxes that offer a female phagostimulants and nutrients, and prevent her from eating the male’s sperm [9, 10]. Using a nutritional geometry framework, researchers showed that decorated cricket males regulate their protein and carbohydrate intake to optimize not only the size of the spermatophylax, but also other fitness-related traits [11]. In many insects, the ingested gift serves a defensive rather than nutritional function. For example, males of the blister beetle *Neopyrochroa flabellate* sequester cantharidin from their diet to gift the female both before (to bias female mate choice) and during copulation; cantharidin serves as a defensive chemical that protects the female and her eggs by making them unpalatable to potential predators [12, 13].

Males of several species of cockroaches offer nitrogen-rich gifts to females, especially in protein-limited environments [14–16]. Males of many species of cockroaches, however, offer a nuptial gift to females in the form of a glandular secretion on the male’s tergum, which he exposes during courtship [17]. The female must mount the male’s dorsum to palpate and feed on this secretion, which places her in a proper position for the male to extend his abdomen and phallomere, grasp her external genitalia, and lock together in a successful mating attempt.

**Fig 1:**
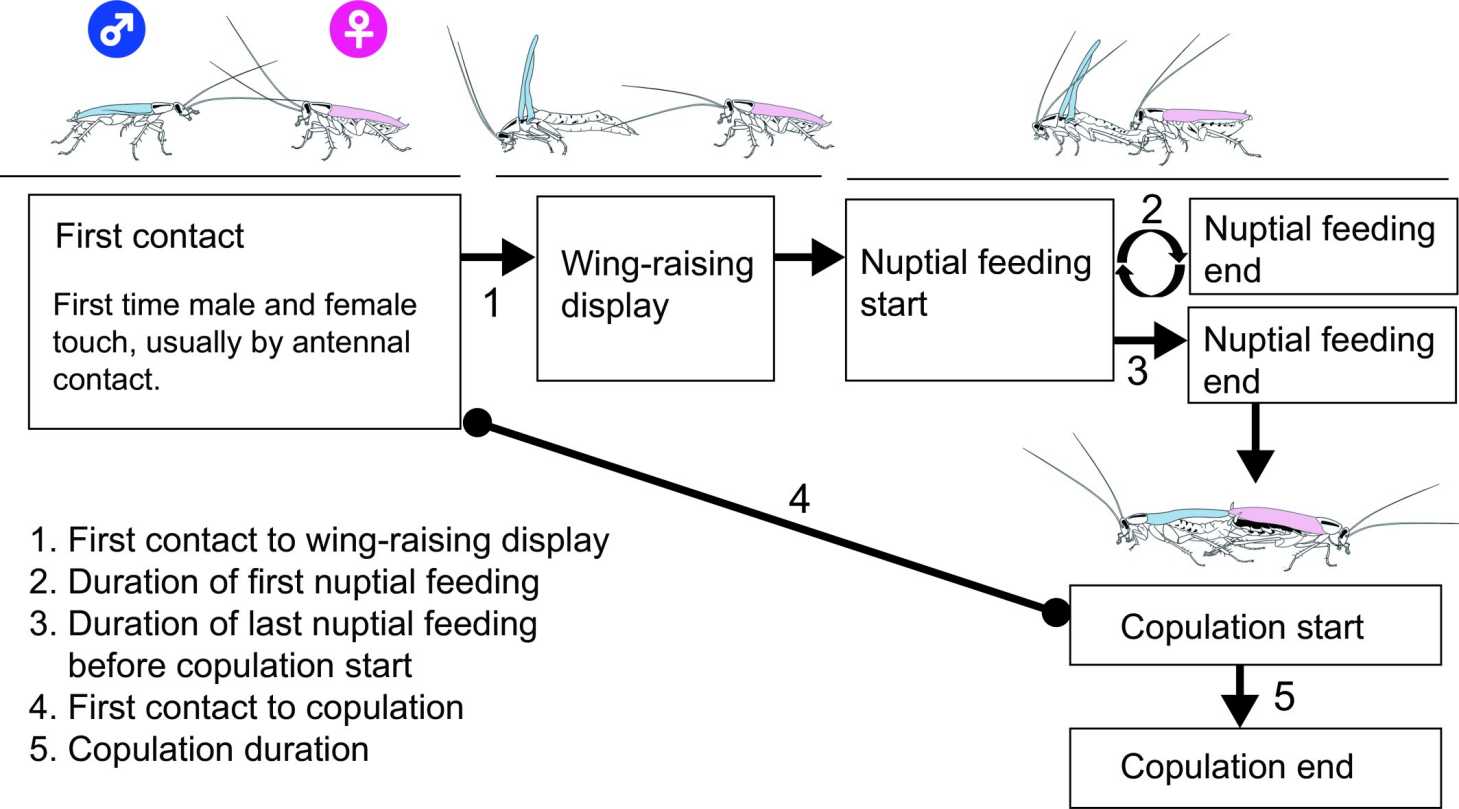
Courtship sequence of the German cockroach. The start and end time of each event was transcribed from video recordings, which were used to calculate latencies and durations of behaviors (denoted 1 to 5). Multiple wing-raising displays and nuptial feeding events may occur between the first and final steps in the sequence.

**Fig 2:**
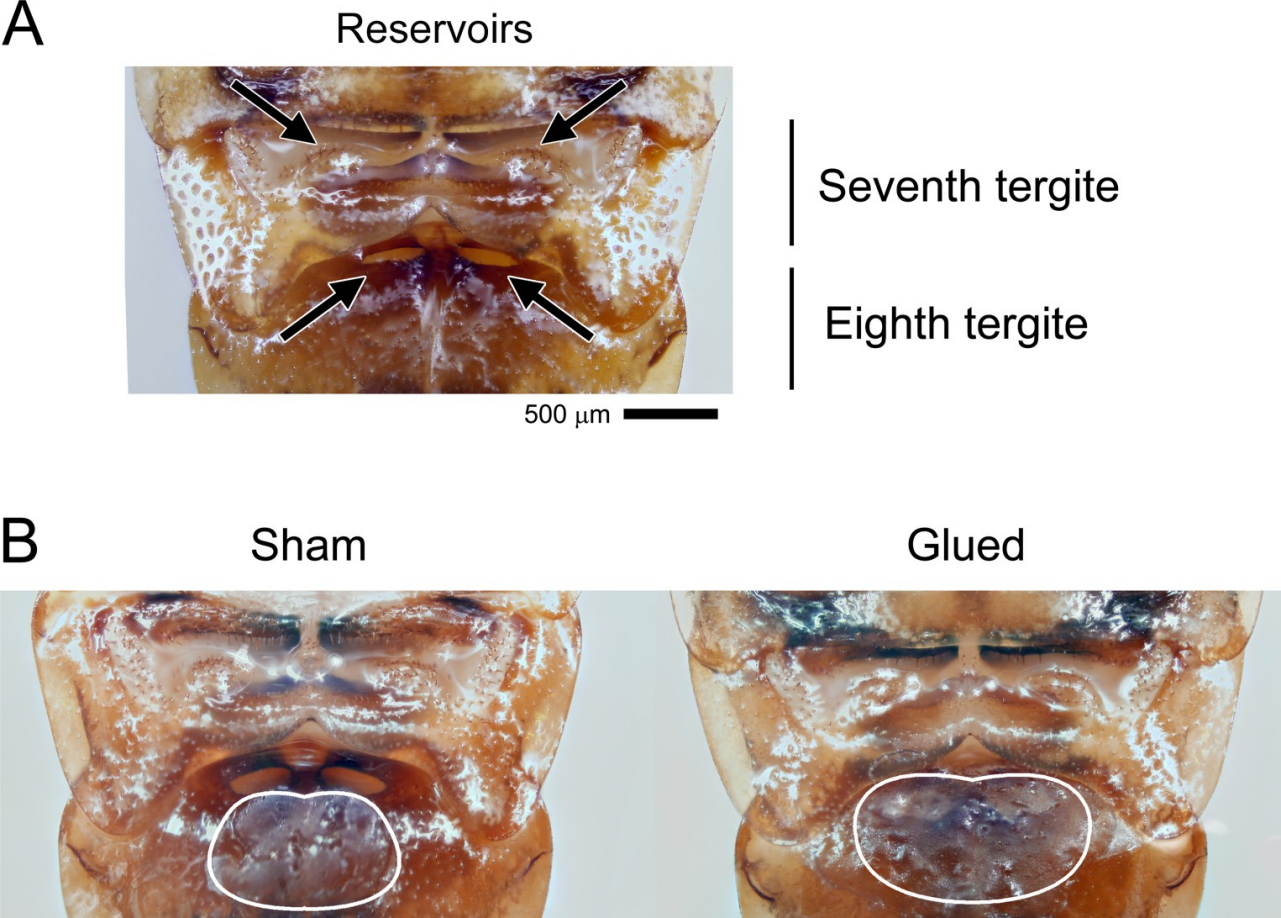
Structure of the male tergal gland and experimental modifications. The unaltered (A) and experimentally modified (B) tergal glands of the male German cockroach. Arrows indicate the slits and wells a female’s mouthparts would contact, located respectively on tergites 7 and 8 (A). Specialized cells secrete gland contents directly into these reservoirs. The wells on tergite 8 are most utilized by females during courtship; they were blocked by glue to determine how ablating this structure would affect mating success. In sham controls, glue was placed near but not covering the wells (B). Gland contents extracted from the reservoirs on tergite 8 were also used for later chemical analysis. Photographs were obtained using an Olympus Digital camera attached to an Olympus CX41 microscope (Olympus America, Center Valley, PA).

The morphology of tergal glands in cockroaches varies from dispersed individual secretory units to highly specialized glands with secretory reservoirs [18]. The male German cockroach (*Blattella germanica*), like many species in the cockroach family Ectobiidae, has a highly specialized tergal gland that plays an integral role in courtship [19]. Sexually receptive females engage in a calling behavior, during which they emit blattellaquinone, a volatile sex pheromone, to attract males [20, 21]. Upon antennal contact (Fig 1), the male perceives a contact sex pheromone on the female cuticle that stimulates him to perform a wing-raising courtship display [22, 23]. The male rotates his body 180^o^, orienting the end of his abdomen toward the female and simultaneously raising his wings [24]. The courtship display exposes the male’s dorsum, including tergites 7 and 8, which house highly specialized tergal glands (Fig 2A). These glands emit as yet unidentified volatile attractants [25]; the sexually receptive female approaches and mounts the male’s dorsum to feed on the glandular secretion (Fig 1). Most of the early feeding activity occurs on reservoirs on the eighth tergite, and the secretion from these reservoirs has been most investigated [21, 26–31].

The unique shape of the tergal gland reservoirs makes it challenging for the female to access the secretion, resulting in longer nuptial feeding [26]. The male senses her position with a specialized sensory structure on the midline of the tergal reservoirs on the seventh tergite [32], which stimulates the male to extend his abdomen and clasp the female’s genitalia with his own. A female can readily reject the male by telescoping her abdomen out of the male’s reach. However, if she accepts the copulatory attempt, nuptial feeding is interrupted as the pair lock genitalia; this ultimately results in the final end-to-end copulatory position, in which they typically remain for approximately 90 minutes [33]. During this time the male transfers sperm in a proteinaceous spermatophore and uses a urate plug to cement the spermatophore in the female’s genital chamber. The spermatophore and urates also serve as a post-nuptial gift–nitrogen-deficient females ingest both and mobilize nitrogen for vitellogenin production [14].

The tergal gland cells of *B*. *germanica* males secrete a synergistically phagostimulatory combination of oligosaccharides and phospholipids [26, 29, 31] directly into the cuticular reservoirs where the female can access it (Fig 2) [34, 35]. The chemical ecology of the phagostimulatory signals has been well investigated, but several features of this mating system have not, and we address them in this report. First, we examined the overall importance of female access to the tergal gland secretion to mating success. Next, we investigated whether the tergal secretion itself was of nutritional value to the female. Then we analyzed mating success and the composition of the tergal secretions of males under different dietary regimes to determine whether the phagostimulatory nature of the secretion varies with male diet.

## Methods

### Insects

Cockroaches were maintained on ad libitum water and rodent diet (Purina 5001 Rodent Diet, PMI Nutrition International, St. Louis, MO) at approximately 27°C and 35% RH on a 12:12 h reversed L:D cycle. All insects were from the Orlando Normal strain, which lacks any insecticide resistance. We separated newly eclosed adults from a cage of late instar nymphs daily in order to have precisely staged cohorts for experiments. Adult females and males were kept in separate incubators until use.

### Role of the tergal gland secretion in courtship behavior

We hypothesized that the male tergal gland secretion and female nuptial feeding are critical to successful courtship. We maintained a group of 60 newly eclosed males for 14 days under the conditions mentioned above. On day 14, we immobilized the insects on ice and separated them into three treatment groups (*n* = 20 each): (a) tergal gland reservoirs blocked with glue (Loctite super glue, Henkel, Ireland), (b) sham-control group with glue near but not blocking the gland reservoirs, and (c) untreated control group (Fig 2). After treatment, there was no male mortality. The photographs of the tergal glands (Fig 2) were obtained using an Olympus Digital camera attached to an Olympus CX41 microscope (Olympus America, Center Valley, PA).

For courtship and mating observations, each sexually mature male was placed in a Petri dish (100 x 15 mm) with a newly eclosed (0-day old) sexually immature female; females become sexually receptive on average 5.7 days after the imaginal molt [33]. Each dish was provisioned with water (in a cotton-stoppered 1.5 ml microcentrifuge tube) and rodent diet. The pair was video-recorded for several days using an infra-red-sensitive camera (Polestar II EQ610, Everfocus Electronics, New Taipei City, Taiwan) interfaced with a data acquisition board and analyzed by searchable and frame-by-frame capable software (NV3000, AverMedia Information, New Taipei City, Taiwan). Recording continued until mating was observed or discontinued after 7 days if the pair failed to mate. We recorded which insects mated or failed to do so.

### Contribution of the tergal secretion to female survival

We hypothesized that the tergal secretion would be of little nutritional benefit to a female, as the volume of the secretion is well below 1 μl [36] and the small reservoir openings prevent females from accessing all of the secretion. Therefore, we investigated whether the nuptial secretion would affect the survivorship of starved females. We fed 0-day-old adult females with rodent diet for four days before separating them into individual 100 x 15 mm Petri dishes and performing one of two treatments on each of them. Each female received two older, sexually mature males (14–24 days) with either intact or glued gland reservoirs on the eighth tergite. Care was taken to avoid the seventh tergite and its associated sensory structure in order to ensure males would behave naturally in response to feeding and feeding attempts. Additionally, the tip of the left phallomere on all males was removed with fine scissors to prevent copulation. This surgical treatment did not affect male courtship behavior. The two males were replaced with fresh males of the same age and treatment daily to control for male starvation. All Petri dishes had a nylon screen at the bottom to minimize coprophagy. Each dish was provisioned with only water, and we compared survival times of females in the two treatment groups.

### Diet treatments of males

We hypothesized that the quality of the male’s diet will affect the tergal gland secretion, courtship, and mating success; we manipulated the male’s diet accordingly. To allow all males to undergo normal sexual maturation, we placed newly eclosed, 0-day-old males in a cage with an egg crate harborage, a cotton-stoppered test tube of water, and rodent diet. They were allowed to develop under normal rearing conditions for 7 days.

On day 7 post eclosion, we separated males into treatment groups. Initially, these groups consisted of males fed for one week either normal rodent diet in agar (nutrient-rich, complete diet) or rodent diet diluted 1:1 with α-cellulose (nutrient-poor diet). When these two groups presented no significant difference in mating success, we switched to more divergent treatments of starved versus fed males. On day 7 post eclosion, we placed individual males in 95 x 15 mm Petri dishes provisioned with water in a cotton-stoppered microcentrifuge tube. The fed males were given ad libitum rodent diet, whereas starved males had water only between days 7 and 14 post eclosion. Housing males individually in dishes prevented cannibalism in the starved treatment.

### Effects of diet treatments on courtship and mating

After 7 days of being either fed or starved, each male was placed in a fresh Petri dish and acclimatized in a temperature-controlled room for a minimum of 15 min in the scotophase. We placed each sexually receptive 5-day-old female into a modified 15 ml polypropylene centrifuge tube. The conical end of the centrifuge tube was sawed off and plugged with cotton. The female was also given at least 15 min to acclimatize in the same room.

We introduced a female to a Petri dish containing a male by slowly sliding the cotton toward the uncapped end of the tube, pushing her into the dish. The pair was then video recorded for at least 15 min, and visually checked every 15 min thereafter for 1 hr to determine mating success. After an hour, we discarded unmated pairs and continued to check mated pairs every 15 min until they separated at the end of mating.

### Nuptial feeding and mating observations

The courtship sequence of *B*. *germanica* is shown in Fig 1. We recorded the time of several courtship and mating events to examine the effects of diet, including:

- Contact: First contact between the female and male.
- Wing-raising display: First wing-raising courtship display by the male.
- Nuptial feeding: The start and end times of the first nuptial feeding event of the female on the male’s tergal gland secretion. In pairs that mated, we also recorded the start and end times of the nuptial feeding event that preceded copulation.
- Mating: The start and end times of copulation. If the pair did not mate within 1 hr they were marked as a failure to mate.

From these events, we obtained five measures of latency or duration: latency from first contact to wing-raising display, duration of first nuptial feeding, duration of the pre-copulatory nuptial feeding, latency from first contact to copulation, and copulation duration.

### Collection of nuptial secretion

We placed five 14-day-old males that had undergone one of the same two treatments used to assess mating behavior (starvation or feeding) in a container (95 × 95 × 80 mm) with 5-day-old females. After each male displayed wing-raising courtship behavior toward the females, we immediately decapitated them and under the microscope drew the nuptial secretion from the gland reservoirs on the eighth tergite into a calibrated borosilicate glass capillary (76 x 1.5 mm). We pooled nuptial secretions from 3 males into each capillary and stored the samples at -20°C until use.

### Sugar analysis using gas chromatography-mass spectrometry (GC-MS)

We focused the GC-MS analysis of male nuptial secretions on maltose and maltotriose. Authentic standards of maltose and maltotriose were diluted in 1 ml HPLC-water and vortexed for 20 sec. We transferred an aliquot of 10 μl of sample to a Pyrex reaction vial containing 10 μl of 5 ng/μl sorbitol (≥98% purity) in HPLC-water as an internal standard; we then dried the samples under a gentle flow of N_2_ for 20 min. We added 15 μl of HPLC-water to each nuptial secretion sample, vortexed it for 30 sec, and centrifuged at 8000 rpm for 5 min to separate lipids from the water layer; we transferred the water phase to a reaction vial using a glass capillary. We repeated this procedure with the remaining lipid layer and then combined the water layers in the same reaction vial containing the sorbitol internal standard and removed the water under N_2_ for 20 min.

For derivatization of sugars, each reaction vial received 12 μl of anhydrous pyridine under a constant N_2_ flow, vortexed and incubated at 90°C for 5 min. We added 3 μl of the silylation reagent *N*-methyl-*N*-(trimethylsilyl) trifluoroacetamide (MSTFA; Sigma-Aldrich, St. Louis, MO) to each reaction vial and centrifuged at 1000 rpm for 2 min. We then incubated vials in a heat block at 90°C for 1.5 hr, vortexing every 10 min for the first 30 min of incubation. The total volume of each sample was ~10 μl.

We injected 1 μl into the GC-MS (6890 GC coupled to a 5975 MS, Agilent Technologies, Palo Alto, CA). The inlet was operated in splitless mode (17.5 psi) at 290°C. The GC was equipped with a DB-5 column (30 m, 0.25 mm, 0.25 μm, Agilent), and helium was used as the carrier gas at an average velocity of 50 cm/s. The oven temperature program started at 80°C for 1 min, increased at 10°C/min to 180°C, then increased at 5°C/min to 300°C, and held for 10 min. The transfer line was set at 250°C for 24 min, ramped at 5°C/min to 300°C and held until the end of program. The MS quadrupole was maintained at 200°C; the ion source was maintained at 70 eV and 230°C. The solvent delay was 9 min, after which the MSD was operated in scan mode with a mass range of 33–650 AMU.

### Statistical analysis

For testing the importance of the tergal gland, we compared percentage mating success according to accessibility of the tergal gland secretion with a Chi-square test (α = 0.05) and applied the Bonferroni method post hoc. To address the nutritional value of the secretion, we used an *F*-test to compare the variances of the two groups, followed by the appropriate *t-*test (assumption of either equal or unequal variance according to the *F*-test result, α = 0.05).

All the behavioral events and latencies were normally distributed, except latency from first contact to wing-raising and latency from first contact to copulation; both were log-transformed. The variance of all measures was analyzed with an *F*-test (α = 0.05) followed by the appropriate two-tailed *t*-test (assumption of either equal or unequal variance according to the *F*-test result, α = 0.05). We also compared mating success according to diet treatment with a Chi-square test (α = 0.05). The GC-MS results were analyzed with the Mann-Whitney *U*-test (α = 0.05).

## Results

### Importance of tergal secretion and nuptial feeding for mating success

**Fig 3:**
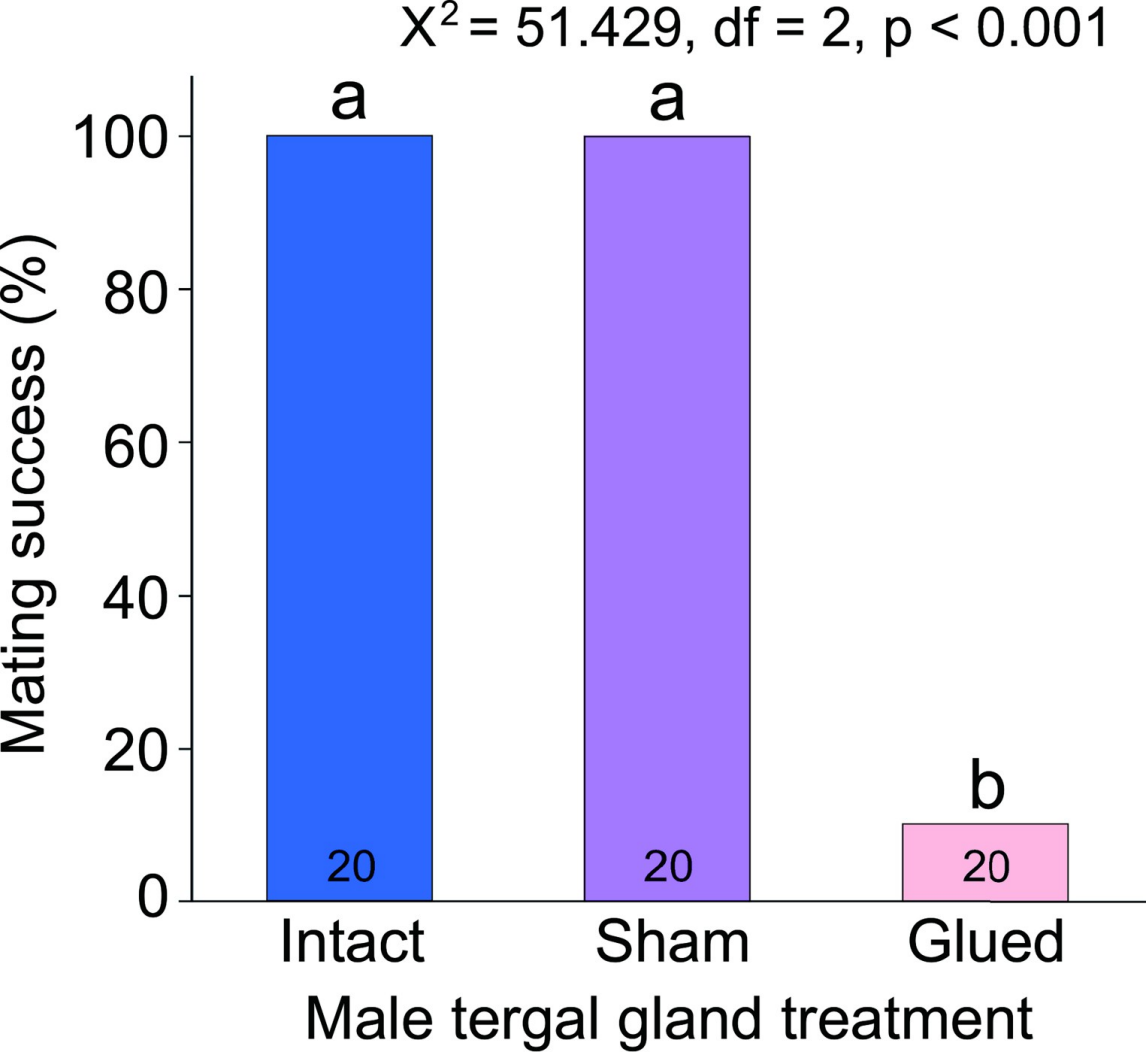
Importance of the male tergal gland in mating success. This experiment consisted of three treatments: males were either unaltered, glued near the tergal gland as a sham-control, or the tergal gland reservoirs on the eighth tergite were occluded with glue (*n* = 20 in each treatment group). The figure displays the percentage of males that mated. We utilized a Chi-square test with a post hoc Bonferroni correction to compare mating success.

To assess the importance of the tergal gland secretion and nuptial feeding in mating success, we occluded the gland reservoirs on the eighth tergite with glue. In the two control groups (untreated and sham-glued) 100% of the males successfully mated (Fig 3). However, when their tergal gland reservoirs were covered with glue, male mating success plummeted significantly by 90% (Χ^2^ = 52.80, df = 1, *p* < 0.001). These results indicate that nuptial feeding by females is a critical event in successful courtship sequences.

### Contribution of the tergal secretion to female survival

**Fig 4:**
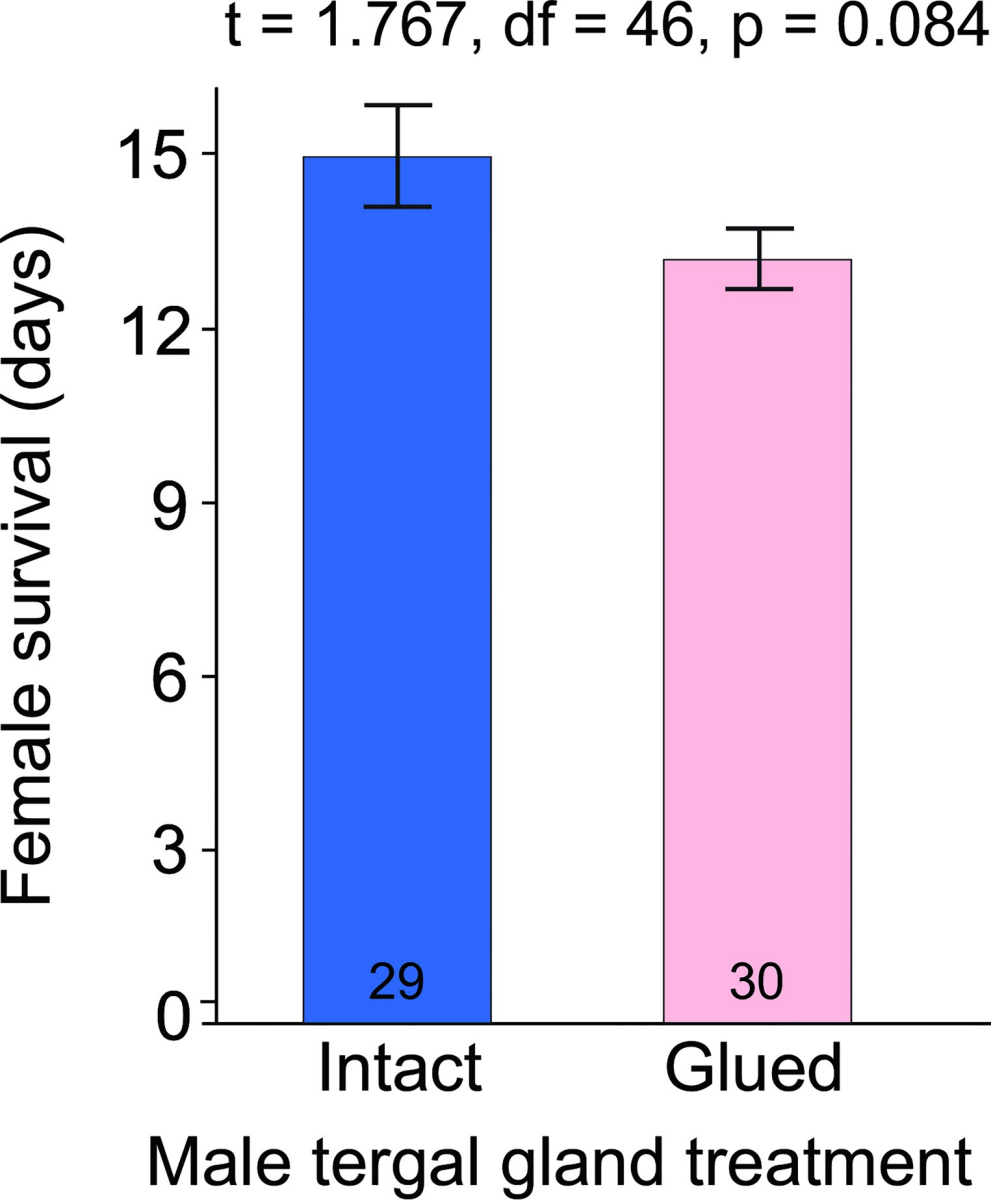
Contribution of the male tergal gland to female survival. Sexually receptive females were given water only and paired with 2 males with intact, untreated tergal gland reservoirs (*n* = 29) or 2 males with their tergal gland reservoirs on the eighth tergite occluded with glue (*n* = 30). Males were phallomerectomized to prevent mating and replaced daily with fresh males of the same respective treatment. Female survival was monitored until all females died, and the treatments were compared with a two-sample *t-*test assuming unequal variances (Welch’s *t*-test).

We evaluated whether the male tergal gland secretion represents only a sexual signal or also a nutritional investment by males. Although females paired with intact males outlived females paired with glued males by 1.8 days (13.6%; 15.0 and 13.2 days, respectively), the *t-*test revealed no significant difference in survival between the two treatments (*t* = 1.767, df = 46, *p* = 0.084, Fig 4). These data, due to unequal variances, necessitated a Welch’s *t*-test (all subsequent data utilized a *t*-test assuming equal variance).

### Mating success and male courtship behavior

**Fig 5:**
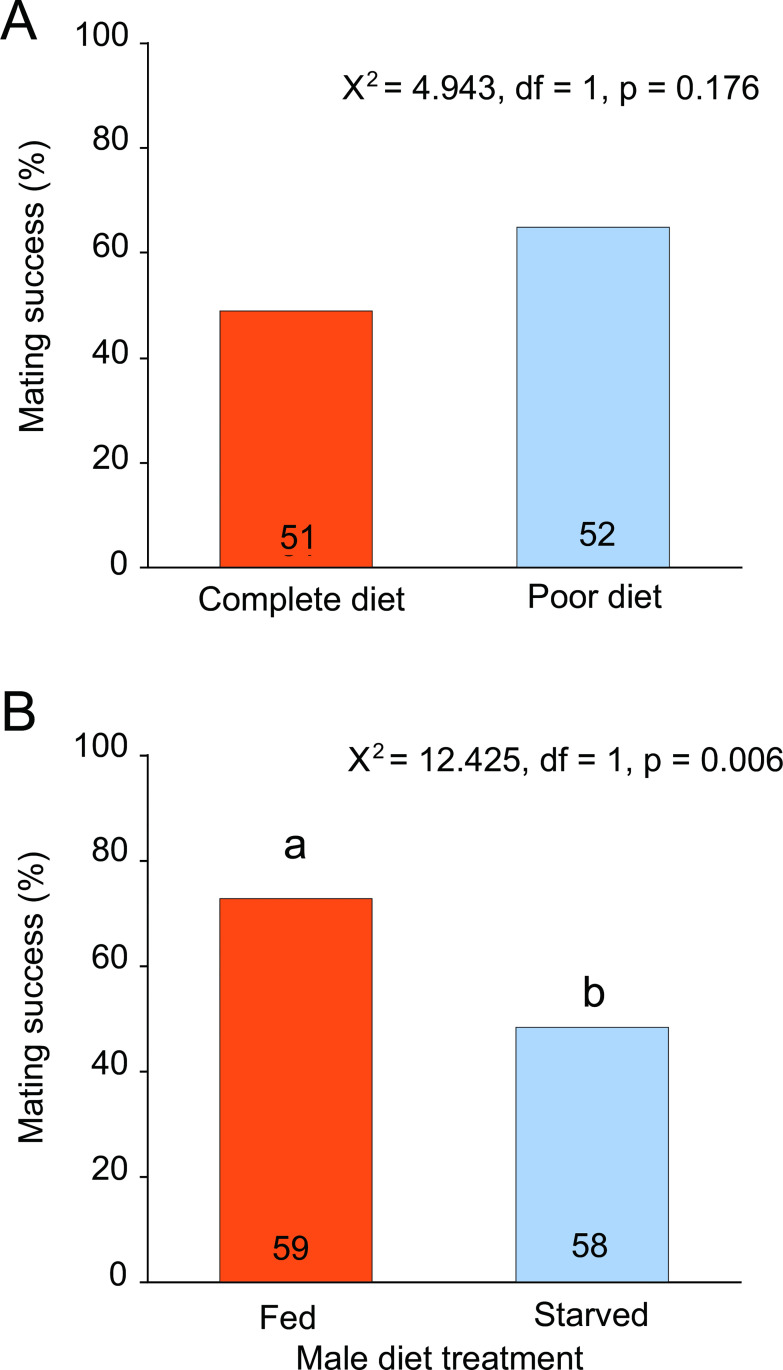
Effects of male nutritional condition on mating success. Pairs that mated within an hour of first contact were considered successful; those that did not were considered a failure to mate. (A) Comparison of mating success of males fed either a nutritionally rich (complete) or nutritionally diluted (poor) diet. (B) Comparison of mating success of fed and starved males; these treatments were used in all subsequent analyses. A Chi-square test was used in both (A) and (B) to compare diet treatments.

We exposed males to complete (nutrient-rich) diets or nutrient-poor diets. However, there was no significant difference in mating success of males fed these two diets (Χ^2^ = 4.943, df = 1, *p* = 0.176, Fig 5A). Therefore, we advanced to a comparison of fed and starved males. Fed males experienced greater mating success (73.9%, *n* = 59) than starved males (48.3%, *n* = 58) (Χ^2^ = 12.425, df = 1, *p* = 0.006, Fig 5B).

**Fig 6:**
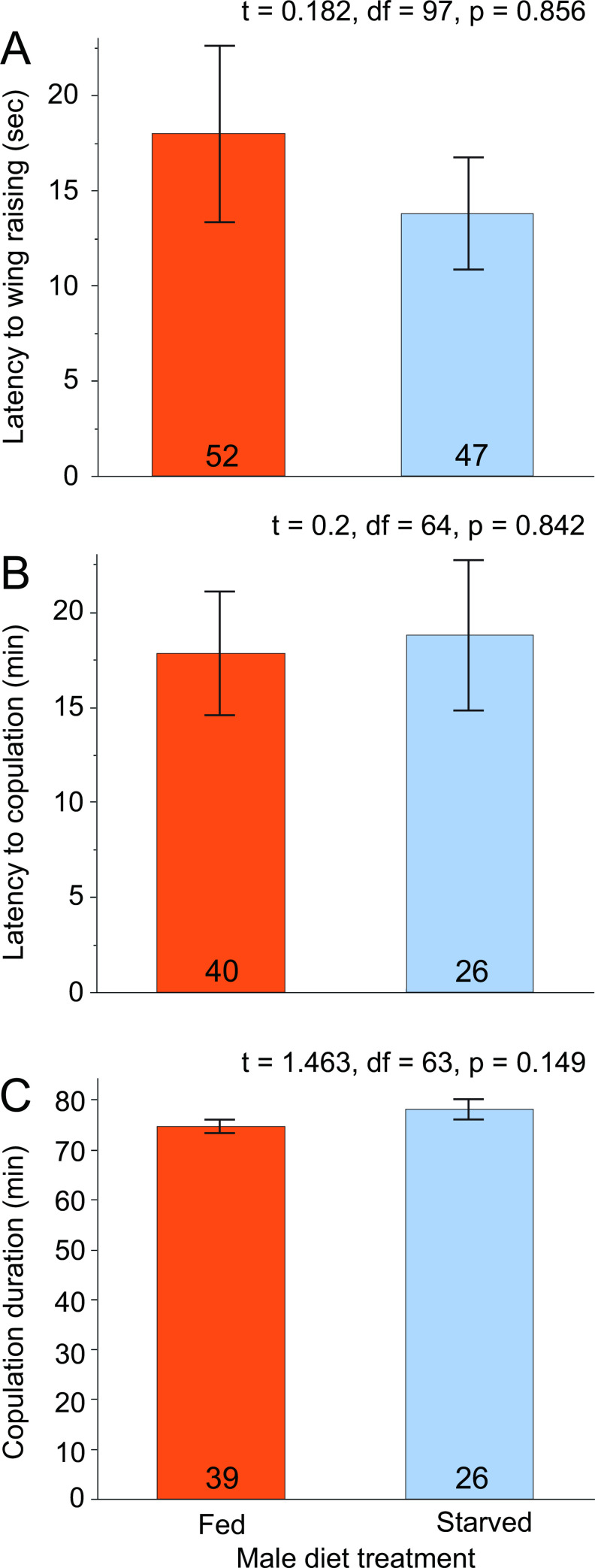
Effects of male nutritional condition on male precopulatory courtship behavior. Three behaviors were examined. The first was the latency from the male’s first contact with the female to the male’s first wing-raising display (A). Latency to copulation is the time between first contact and successful copulation (B). Duration of copulation was from coupling to separation of the pair (C). For each assay, fed and starved males were compared using a two-sample *t*-test assuming equal variance.

We sought to determine whether differences between fed and starved males could be attributed to differences in their courtship behavior. The data for latency from first contact between the male and female to wing-raising display necessitated a log-transformation to normalize the distribution. The mean times (latencies) taken by the fed and starved males to wing-raise after their first contact with a female were not significantly different (*t* = 0.182, df = 97, *p* = 0.856) (Fig 6A).

The latency from first contact to successful copulation also required a log-transformation to normalize the data. Latency to copulation was again not significantly different between starved and fed males (*t* = 0.200, df = 64, *p* = 0.842, Fig 6B).

Although starved males coupled longer (78.2 min, *n* = 26) than fed males (74.8 min, *n* = 39), this difference was not significant (*t* = 1.463, df = 63, *p* = 0.149, Fig 6C). Overall, it appeared that starved and fed males exhibited similar latencies to courtship events and similar copulation durations, suggesting that male courtship behavior was unaffected by the diet treatments, and that mating success might instead be affected by female behaviors.

### Nuptial feeding

**Fig 7:**
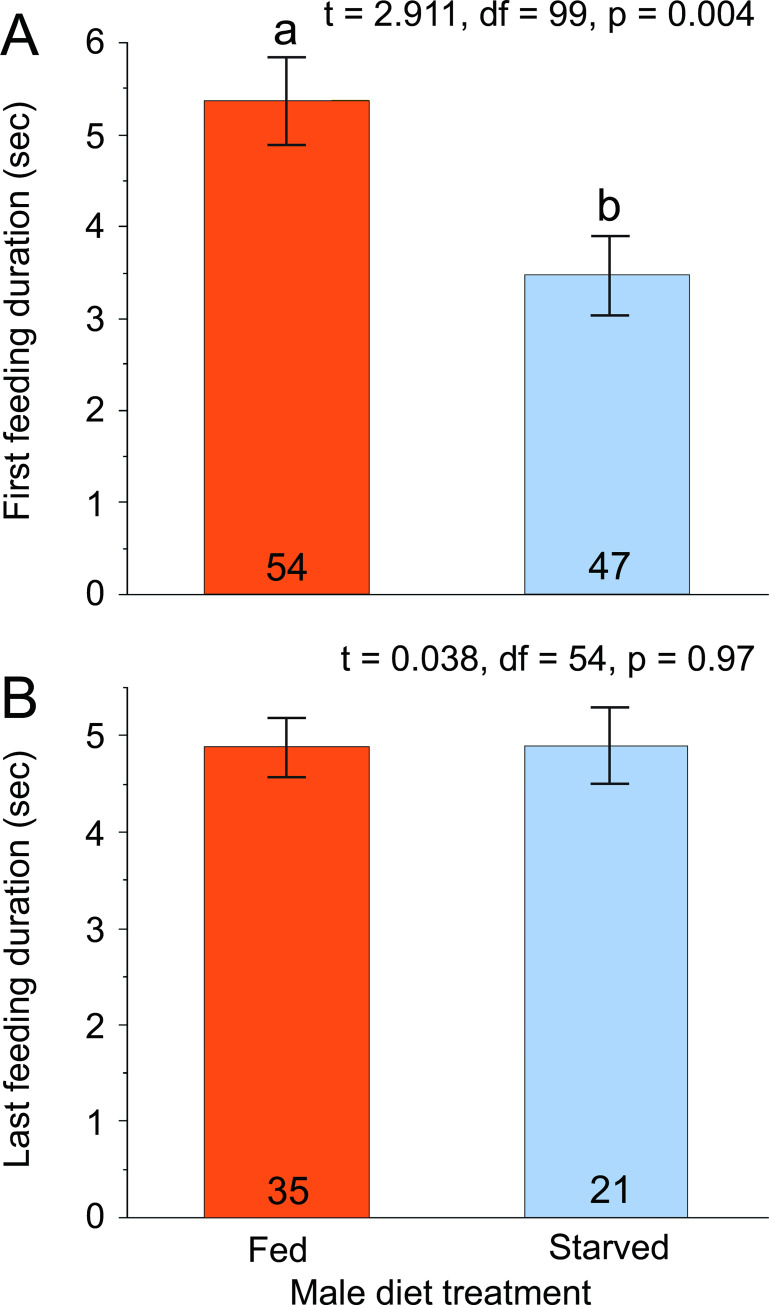
Effects of male nutritional condition on female nuptial feeding on the tergal gland secretion. The first nuptial feeding event did not lead to a successful mating for any males (A). The last nuptial feeding event measures the duration of the final feeding event that was followed by a successful mating (B). For each assay, fed and starved males were compared using a two-sample *t*-test assuming equal variance.

In their first nuptial feeding, which did not lead to copulation, sexually receptive 5-day old females fed significantly longer on the tergal gland secretion of fed males (5.4 sec, *n* = 54) than unfed males (3.5 sec, *n* = 47) (*t* = 2.911, df = 99, *p* = 0.004, Fig 7A). In contrast, the duration of the last nuptial feeding event that led to successful copulation was not significantly different for fed (4.9 sec, *n* = 35) and starved (4.9 sec, *n* = 21) males (*t* = 0.038, df = 54, *p* = 0.970, Fig 7B). The sample size for the first (unsuccessful) feeding event was roughly double that of the last (successful) feeding. This discrepancy was due to (a) the last feeding occurring only in successfully mated pairs, and (b) many pairs mating after the 15 min video recording, resulting in the last tergal feeding not being recorded for these pairs.

### Chemical analysis of the tergal gland secretion

**Fig 8:**
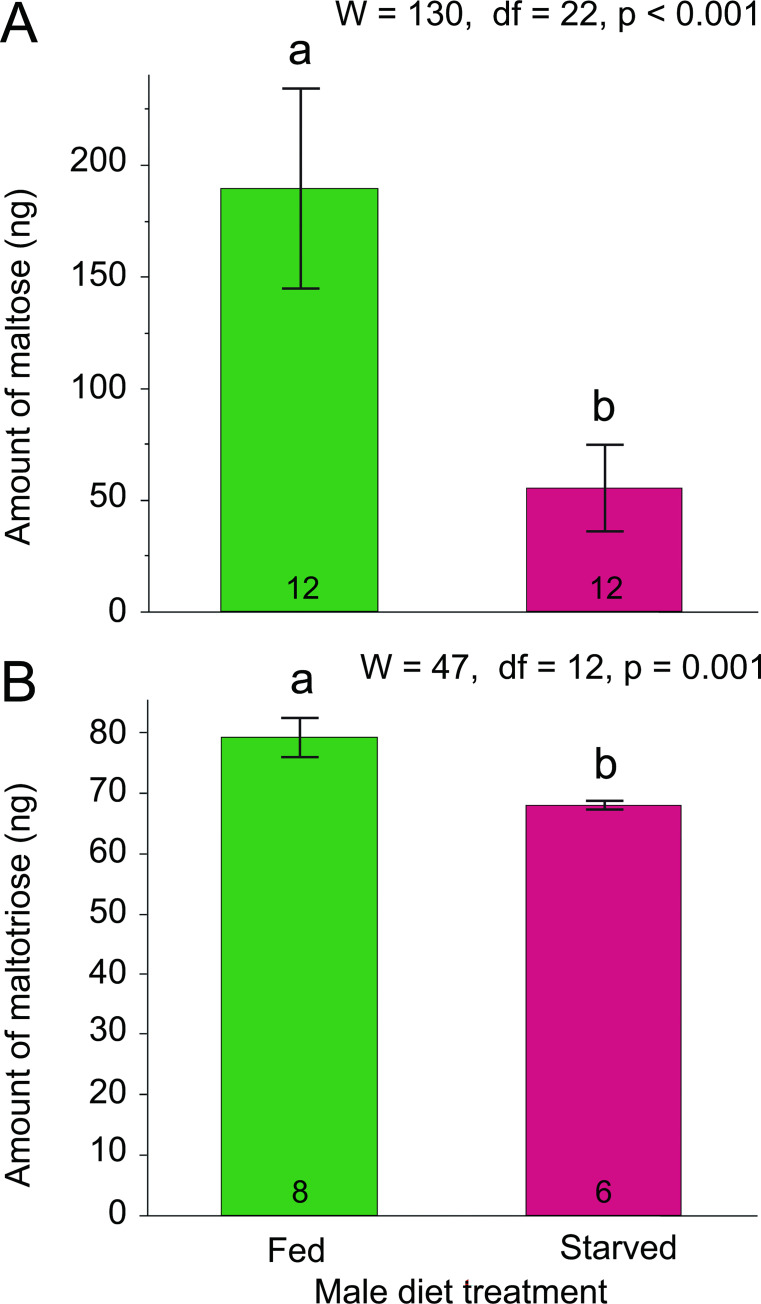
Effect of male nutritional condition on sugars in the nuptial secretion. Maltose (A) and maltotriose (B), both major oligosaccharides in the tergal gland, were used to measure the effect of diet on the tergal gland secretion. Treatments were compared for each sugar using a Mann-Whitney *U*-test.

Chemical analysis of tergal gland contents from starved and fed males showed significantly higher levels of phagostimulatory sugars in the fed males (Mann-Whitney *U*-test, maltose: W = 130, df = 22, *p* <0.001; maltotriose: W = 47, df = 12, *p* = 0.001, Fig 8).

## Discussion

In this investigation, we explored whether the tergal gland secretion that German cockroach males offer to females during courtship is required for successful mating. The results showed that 100% of the males with exposed gland reservoirs mated, whereas only 10% of males with occluded gland reservoirs did so. From these results, we infer that features of the tergal gland (such as location, morphology, chemistry) are under strong selection to optimally position and arrest female movement long enough for the male to successfully copulate with her. We also tested the hypothesis that the tergal gland secretion represents not only a phagostimulatory signal, but a male nutritional investment as well. Our results support the phagostimulatory nature of the secretion. However, despite access to the secretion extending survival of starved females by 1.8 days, we reject the idea that the male’s secretion would be of significant nutritive value to females under normal field conditions.

We also tested the hypothesis that the vital function of the tergal gland secretion is affected by the nutritional state of the male. Significantly fewer starved than fed males mated successfully, indicating that some characteristic(s) of nutritionally deficient males hindered mating. After dissecting the events of the courtship sequence, we found no differences between starved and fed males in male-controlled behaviors, including latency to the first courtship display, latency to copulation, and duration of copulation.

Next, we examined female-controlled behaviors. In a recent study we showed that nuptial feeding duration is a critical parameter that determines mating success [36]. The first courtship and the nuptial feeding that followed were always unsuccessful, so we used it to detect differences between starved and fed males. Females spent significantly more time feeding on the tergal secretion of fed males than starved males. The last nuptial feeding event that resulted in copulation was not significantly different on fed and starved males. We inferred from these results that females sensed a deficiency in the tergal secretion of starved males and so engaged in shorter nuptial feeding. Finally, quantitative analysis of sugars in the tergal gland reservoirs of fed and starved males confirmed that titers of both maltose and maltotriose were significantly lower in starved than in fed males. We therefore conclude that the quality of the tergal gland secretion (i.e., sugar content and possibly other components) is affected by the male’s nutritional status. This secretion may inform females of male quality and feature prominently in female mate choice.

### Sensory bias and evolution of the tergal gland signal as a sensory trap

The male’s tergal secretion might have evolved as sensory exploitation of the female’s pre-existing sensory bias; it fits the definition of adaptive sensory bias, where females evolve under natural selection to respond to adaptive stimuli and males subsequently evolve novel sex-specific traits under sexual selection that target the female’s sensory biases [37, 38]. The female stimuli and male adaptations are in this case specific sugars and tergal gland secretions, respectively. The onset of sexual receptivity in females corresponds to a stage of rapid oocyte maturation during which females consume large amounts of foods that support vitellogenin production [39, 40]. Females at this stage are therefore highly motivated to feed and have low response thresholds for acceptance of phagostimulatory sugars [31].

The male appears to exploit this pre-existing gustatory bias by offering the female a sugar-rich nuptial secretion. Maltose and maltotriose, as well as longer glucose-based oligosaccharides, are prominent in the tergal gland secretion [27] and are highly phagostimulatory to females [28], even at relatively low concentrations [31, 41, 42]. In addition to sugars, the tergal secretion contains phospholipids that synergize the electrophysiological responses of the peripheral gustatory neurons and behavioral responses of the female [31].

In this way the male courtship signal appears to not only mimic stimuli that females use in foraging contexts, but the combination of sugars and phospholipids may also represent a more effective (super normal) stimulus relative to the food models it mimics. Based on these characteristics we infer that the nuptial secretion likely evolved as a sensory trap–the male utilizes a blend of phagostimulants to co-opt the sense of taste, a chemosensory signal and sensory modality that functions in contexts other than courtship; successful mating closely depends on the quality of the male signal [43].

Notably, under the sensory bias hypothesis, female preferences predated the evolution of sexually dimorphic male nuptial stimuli. However, while female sensory preferences can rapidly evolve (e.g., through changes in olfactory and gustatory receptors), compensatory evolutionary changes in the male’s tergal secretion may be slower. Thus, adaptive sensory bias may impose mismatches between the female’s gustatory preferences and the male’s courtship signals that evolved to exploit the female’s preferences. Recently, we discovered such a chemosensory divergence in the German cockroach. In response to human-imposed selection with sugar-containing insecticide baits, cockroach populations evolved glucose-aversion, a highly adaptive behavior driven by changes in gustatory neurons housed in sensory sensilla on the mouthparts [44]. However, glucose-averse females experience lower mating success because salivary glucosidases rapidly digest the male’s nuptial sugars into glucose, which deters glucose-averse females during courtship [36]. Thus, male courtship signals that evolve under strong sexually selected sensory bias are susceptible to disruption when female chemosensory preferences adaptively diverge under natural selection.

### Functions of the tergal gland secretion

A highly specialized tergal gland is a male-specific adult trait in cockroaches. Its architecture, chemistry, and pivotal role in successful courtship suggest that this gland is under strong selection by females. We considered three potential functions of the tergal gland secretion. The first is to arrest the female for copulation. Our recent findings indicate that long nuptial feeding is associated with greater mating success; conversely, interrupted, short nuptial feeding events result in failed courtships [36]. The presence of phagostimulatory compounds and the morphology of the tergal gland openings prolong nuptial feeding and increase the probability of mating.

Its second function might be to provide females a nutritional resource. German cockroach females invest large amounts of nutritional resources and time into each clutch of eggs. Every ootheca contains approximately 40 individual embryos and weighs approximately 60% of the female body mass [45]. The female carries the egg case throughout embryogenesis; therefore, for approximately three weeks this “gestation” period prevents the female from provisioning a new clutch of oocytes. This strategy contrasts with other oviparous cockroaches, most of which deposit their oothecae, leaving them more vulnerable to desiccation, parasitoids, and other environmental threats. A gravid female (i.e., one carrying an extruded egg case) eats and drinks much less than females that are not so encumbered [45], so it is especially important for her to stockpile nutrients to prepare for this lean three-week period. If she dies during this time, or prematurely drops the ootheca, the embryos may desiccate and perish [46].

The gland reservoirs on the eighth tergite have small openings which limit the female’s access to the secretion. Only the gustatory sensilla on the female’s maxillary and labial palps can sample the secretion, and precise maneuvering is required for the female to taste the secretion with her paraglossae and ingest it [26, 31]. The tergal gland reservoir contains only ~50 nl of a viscous liquid secretion [36]; females are capable of drinking up to 3 μl of water and consuming >10 mg of food per day [40]. Therefore, it would appear that the tergal gland secretion would not represent a resource-rich nuptial gift. Our empirical evidence confirmed that continuous access to tergal gland secretions had little effect on the survival of starved females, due to either its minimal volume or limited availability. This conclusion is consistent with findings in crickets, where “the spermatophylax appears to be more of a sham than a true gift”, and “there was no effect of spermatophylax consumption on female survival, egg size, or lifetime reproduction, even when females were completely deprived of food” [10]. However, as Sakaluk et al. [10] point out, the production of the male offering may still incur significant costs and thus may reflect a male trait that females prefer.

A possible third function of the tergal gland secretion might be as an honest signal of male quality and his potential reproductive fitness. In *B*. *germanica*, females have multiple checkpoints to assess the male. Precopulatory checkpoints include his courtship behavior and quality of the nuptial secretion. Post copulation, the size of the spermatophore may come into play; small spermatophores are less effective at suppressing sexual receptivity of female cockroaches [47], which could result in sperm competition and sperm displacement. The male also provisions the female with a urate plug that serves as a nitrogen investment. Both post-copulation gifts are much more substantial than the courtship secretion [14–16, 48, 49] and may allow the female to adjust her precopulatory assessment of the male. It makes sense for the male to present the female with an honest nuptial gift that informs her of the quality of the post-copulatory gift; dishonesty could result in later rejection of his sperm, rendering the whole mating endeavor a waste of valuable resources.

The associations between the spermatophore, urates, and tergal gland secretion may be linked to the male’s dietary intake and nutritional status. It is noteworthy in this regard that cockroach species with highly specialized tergal glands (Ectobiidae) also have highly developed uricose glands that deliver post-copulatory urates to females [19, 50]. The association of tergal gland secretions with diet quality could signal to the female the male’s foraging success, a potentially heritable trait, and thus overall male fitness. The diet may also affect sperm quality and therefore female fecundity. German cockroaches typically mate once and store sperm in a spermatheca for fertilization of subsequent oocytes [51]. Therefore, indirectly assessing male (and sperm) quality through the quality of the nuptial secretion could have long-term implications to reproductive fitness. It is important to note that the function of the tergal gland secretion as an honest signal is not inconsistent with it also evolving as a sensory trap. There is evidence that in some species a sensory trap may over time become an honest signal as males invest greater resources in nuptial and post-copulatory gifts, especially when females exercise cryptic mate choice [52].

### Follow-up questions: Composition of the nuptial secretion and female receptivity

With the revelation that nutrition affects the quality of a male’s tergal gland secretion, and consequently mating success, several follow-up questions should be considered. Nutrition studies with the German cockroach have typically focused on their protein and carbohydrate intake under laboratory conditions [53, 54], although some have suggested that fats may be important in the field [55]. Previous findings have shown that males increase protein consumption the more they mate [53] and that cockroaches in different homes prefer different nutrient ratios [56]. Since the tergal secretion contains a synergistic blend of oligosaccharides, phospholipids, and proteins [29, 31], it might represent an optimal nutritional mix that females prefer. Further metabolomic and proteomic studies are warranted to elucidate the composition of the tergal secretion. It might inform a fundamental understanding of cockroach gustatory preferences.

Sexual receptivity of *B*. *germanica* females varies throughout the gonadotrophic cycle, as does her intake of nutrients and preferences for specific foods and protein-to-carbohydrate ratios [20, 33, 39, 40, 54, 57]. Our study utilized females of a known age fed a nutritionally complete diet to control for these factors, but the nutritional state of sexually receptive females may vary considerably in the field. Once the effect of male diet on the tergal gland is established, future studies should vary female nutrition. It would be fascinating to know whether the male’s tergal gland contents are optimized for the gustatory preferences of females of various nutritional states, and if the male can alter his secretion depending on the local nutritional landscape that females and males share. It may also be worthwhile to observe how females exercise choice when presented with multiple males. While it is unclear how often two or more males directly compete for a single female *in situ*, one cannot dismiss the possibility, especially in large aggregations.

Understanding the interplay between female preferences, male courtship stimuli, and the effects of nutrition on both could ultimately provide a more robust understanding of this crucial sexually selected male trait, and perhaps some insight into its evolution and that of similar traits in other taxa.
